# The role of glycation in the pathogenesis of aging and its prevention through herbal products and physical exercise

**DOI:** 10.20463/jenb.2017.0027

**Published:** 2017-09-30

**Authors:** Chan-Sik Kim, Sok Park, Junghyun Kim

**Affiliations:** 1. Korean Medicine Convergence Research Division, Korea Institute of Oriental Medicine, Daejeon Republic of Korea; 2. Department of Sports Leadership, Kwangwoon University, Seoul Republic of Korea; 3. Department of Oral Pathology, School of Dentistry, Chonbuk National University, Jeonju Republic of Korea

**Keywords:** Advanced glycation end products, Aging, Glycation, Herbal products, Physical exercise

## Abstract

**[Purpose]:**

Advanced glycation end products (AGEs) are non-enzymatic modifications of proteins or lipids after exposure to sugars. In this review, the glycation process and AGEs are introduced, and the harmful effects of AGEs in the aging process are discussed.

**[Methods]:**

Results from human and animal studies examining the mechanisms and effects of AGEs are considered. In addition, publications addressing means to attenuate glycation stress through AGE inhibitors or physical exercise are reviewed.

**[Results]:**

AGEs form in hyperglycemic conditions and/or the natural process of aging. Numerous publications have demonstrated acceleration of the aging process by AGEs. Exogenous AGEs in dietary foods also trigger organ dysfunction and tissue aging. Various herbal supplements or regular physical exercise have beneficial effects on glycemic control and oxidative stress with a consequent reduction of AGE accumulation during aging.

**[Conclusion]:**

The inhibition of AGE formation and accumulation in tissues can lead to an increase in lifespan.

## INTRODUCTION

Aging is defined as a progressive loss of the efficacy of biochemical and physiological processes that occur until death^1^. A number of theories have been introduced to explain the aging process. One theory is that the abnormal accumulation of biological waste products in the organism is responsible for organ or tissues senescence^2, 3^.

Glycation is a spontaneous non-enzymatic reaction of free reducing sugars with free amino groups of proteins, DNA, and lipids that forms Amadori products. The Amadori products undergo a variety of irreversible dehydration and rearrangement reactions that lead to the formation of advanced glycation end products (AGEs). This process was first introduced by Louis-Camille Maillard in 1912^4^. The glycation process leads to a loss of protein function and impaired elasticity of tissues such as blood vessels, skin, and tendons^5-7^. The glycation reaction is highly accelerated in the presence of hyperglycemia and tissue oxidative stress^8^. This implicates it in the pathogenesis of diabetic complications and aging^9^. Because there are no enzymes to remove glycated products from the human body, the glycation process matches well with the theory that the accumulation of metabolic waste promotes aging.

Oxidative stress has a very important role in the mechanism by which AGEs form and accumulate, and has been implicated as a key factor in the progression of various diseases, including chronic diseases such as diabetes, Alzheimer's disease, and aging^10-12^. Oxidative stress, more specifically oxidative damage to proteins, is increasingly thought to play a central mechanistic role in this context, as it is associated with modifications in the activities of biological compounds and cellular processes that may be linked to a pathological environment. Oxidative stress is fueled by the generation of excessive reactive oxygen species (ROS) from glucose autoxidation, and also the nonenzymatic, covalent attachment of glucose molecules to circulating proteins that result in the formation of AGEs^13^.

Naturally occurring phytochemicals and products are relatively safe for human consumption as compared to synthetic compounds, and are relatively inexpensive and available in orally ingestible forms. The search for an inhibitor of AGE formation has identified several natural products that prevent the glycation process. A number of medical herbs, dietary plants, and phytocompounds inhibit protein glycation both in vitro and in vivo^14^. These natural products with high antioxidant capacity may be promising agents for the prevention of glycation and AGE formation. Their anti-AGE activity may be one mechanism of their beneficial actions on human health^15^.

Numerous previous reports indicate that the gradual decrease in systemic antioxidant capacity is the casuse of biological aging^16^. Other evidence supports the wide consensus that physical exercise improves systemic antioxidant activity^17^. Physical exercise can decrease oxidative stress in rodent animal models^18, 19^. Moderate physical exercise induces the expression of antioxidant enzymes, leading to the reduction of oxidative stress^20^. Additionally, regular physical exercise reduces AGE levels in renal tissues of obese Zucker rats^21^ and has a beneficial effect on glycemic control in patients with diabetes^22^. Therefore, physical exercise may be a powerful weapon against AGE formation and AGE-related aging processes.

In this review, we discuss the implication of AGEs on the aging process. We also consider the potential inhibitory activity of herbal products and physical exercise in age-related organ dysfunction induced by glycation and/or AGEs, and the underling mechanisms.

## DEFINITION of GLYCATION and AGEs

**Figure 1.**
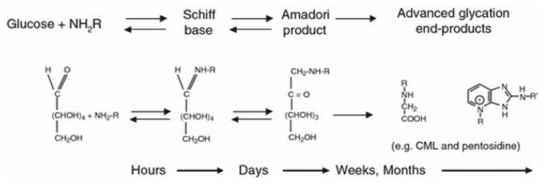
Glycation process leading to the formation of advanced glycation end-products (AGEs). Illustration from Bohlender et al., 2005.

**Figure 2.**
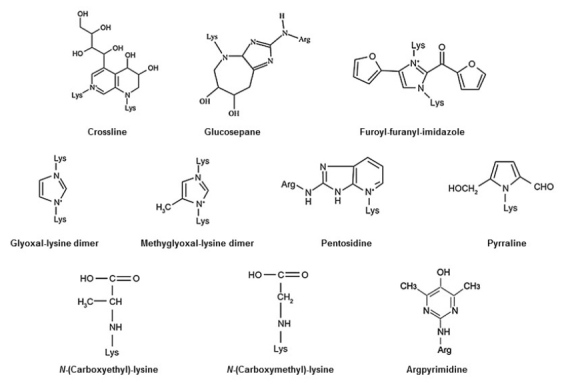
Examples of biologically relevant advanced glycation end-products (AGEs). Illustration from Sadowska-Bartoz and Bartosz, 2016.

AGEs were initially identified in the cooking process as the result of a nonenzymatic reaction between sugars and proteins within foods; this reaction is called the Maillard reaction^4^. The glycation process is initiated by a chemical reaction between the reactive carbonyl group of a sugar or an aldehyde with a nucleophilic free amino group of a protein, leading to the rapid formation of an unstable Schiff base. This adduct then undergoes rearrangement to form a reversible and more stable Amadori product. These intermediate products undergo further irreversible oxidation, dehydration, polymerization, and cross-linking reactions resulting in the formation of AGEs over the course of several days to weeks (Figure 1). Some important AGE compounds are shown in Figure 2.

## ROLE of AGEs DURING AGING

The accumulation of glycated macromolecules, including proteins, is a hallmark of aging both in humans and experimental animals. The accumulation of AGEs was shown in Drosophila melanogaster and Caenorhabditis elegans. The content of AGEs in young (10 days old) D. melanogaster flies is 44% lower than in senescent (75 days old) flies^23^. C. elegans grown under high glucose conditions (40 mM) have a shortened lifespan and increased AGE content^24^. Table 1 shows the available evidence for the accumulation of AGEs during aging and in different pathologies.

**Table 1.** AGE accumulation in tissues during aging.

Tissue	AGEs	Commentary	Reference
Heart	CML	Increase with age	68
Lamina cribrosa	Pentosidine	Increase with age	69
Lung collagen	Pentosidine	Increase with age	70
Patellar tendon	Pentosidine	Increase with age	62
Skin	Argpyrimidine Pentosidine	Increase with age	71
Vitreous body	Pentosidine	Accumulation with age	72
Oocytes	Pentosidine	Increase with age	73
Intervertebral disk	Pentosidine	Increase with age	74
Cartilage	Pentosidine CEL, CML	Increase with age	75

CEL, *N*-(carboxyethyl)-lysine; CML, *N*-(carboxymethyl)-lysine.

Glycation is one of the endogenous aging mechanisms that occurs spontaneously with time, but also in a pathological manner during diabetes, renal failure, and inflammation^25^. AGEs are highly accumulated in tissues and organs in numerous age-related degenerative diseases. These toxic adducts (glycotoxins) are implicated in cell dysfunction, especially in diabetic patients and older organisms. AGE formation and accumulation in diabetic patients results in vascular alterations leading to diabetic vasculopathy.

There are three major mechanisms by which AGEs induce injury to the extracellular matrix (ECM) and cells, thereby contributing to aging and age-related diseases: (1) accumulation of AGEs within the ECM (such as collagen and elastic fibers) and cross-linking between AGEs and ECM causing a decrease in connective tissue elasticity, (2) glycated modifications of intracellular proteins causing a loss of the original cellular function, and (3) interaction of AGEs with their cellular receptor (RAGE), leading to the subsequent activation of inflammatory signaling pathways, ROS generation, and apoptosis^26^.

Glycation of extracellular proteins induces the cross-linking of collagen and elastic fibers. As a consequence, elasticity of the ECM is altered, affecting especially vascular functions. There is a marked correlation between the serum concentration of N-(carboxymethyl)-lysine (CML) and vessel stiffness in elderly individuals^27^. Altering the balance between synthesis and degradation of ECM by glycated modifications may accelerate skin aging and increase skin stiffness^28^. Furthermore, cross-linking between AGEs and collagen impairs the mechanical properties of collagen. In particular, the cross-linking of AGEs with collagen of the vascular wall alters its structure and function, facilitating plaque formation and basement membrane hyperplasia^29^.

Glycation also affects intracellular proteins. Intracellular AGE-modification of signaling molecules may impair cellular functions and gene expression^30^. For example, the activities of several antioxidant enzymes, including catalase, glutathione peroxidase, and glutathione reductase, are reduced by glycated modifications. Alterations of these enzyme activities increases cellular oxidative stress^31, 32^. In addition, glycated proteins are usually removed via ubiquitin-dependent 20S proteasome-mediated proteolysis. AGE-modifications can disturb this proteolytic degradation, contributing to a further increase in the cellular content of glycated proteins^33^.

RAGE is the best-characterized cell surface molecule that recognizes AGEs. The interaction between an AGE and its receptor alters cell and organ functions mainly through inflammatory molecules, leading to aging. RAGE regulates a number of cell processes of crucial importance such as inflammation, apoptosis, ROS signaling, proliferation, autophagy, and aging^34, 35^.

## DIETARY AGEs

Endogenous glycation reactions occur spontaneously with a small proportion of intestinally absorbed sugars^36^. However, food is an important source of exogenous AGEs. The role of dietary AGEs and their interaction with RAGE during aging has been demonstrated recently^37^. The Maillard reaction is often used to improve the color, flavor, aroma, and texture of foods. However, significant generation of AGEs occurs when sugars are cooked with proteins^38^.

In a mouse model, feeding an AGE-rich diet for 16 weeks promoted a 53% increase in the serum levels of AGEs^39^. Uribarri et al. reported that, in renal failure patients, there was a 29% increase in CML levels in the blood of those subjected to an AGE-rich diet, while a 34% reduction of CML was detected in the group fed a low AGE diet^40^. In a mouse model, a 9-month dietary exposure to CML accelerated endothelial dysfunction and arterial aging. These results suggest that a diet restricting AGEs could be an effective way to reduce the AGE burden in the human body.

## AGE INHIBITORS

There is considerable interest in the therapeutic potential of agents that can inhibit the formation of AGEs or break AGE-mediated cross-links^41, 42^. Several synthetic or natural agents have been proposed as AGE inhibitors.

Aminoguanidine was first introduced as an AGE inhibitor^43^. AGE inhibitors, including aminoguanidine and pyridoxamine, prevent AGE accumulation by interacting with the highly reactive carbonyl species and acting as carbonyl traps^44, 45^. In previous reports, aminoguanidine prevented diabetic renal, retinal, and neural complications through the inhibition of AGE formation^46^. However, due to safety concerns resulting from its adverse effects, including pro-oxidant activities^47^ and inhibition of NO synthase^48^, aminoguanidine cannot be used clinically^49^.

Recently, several researchers have suggested that a novel agent can destroy preformed AGE-derived protein cross-links. The first identified AGE breaker, N-phenacylthiazolium bromide, was introduced in 1996. Because N-phenacylthiazolium bromide is unstable in vitro, it was not clinically successful. Another compound, alagebrium^50^, was developed as an AGE breaker. Alagebrium could reverse AGE accumulation in vivo^51^. However, clinical studies on these compounds were terminated and none of the known AGE breakers are in clinical use.

Herbal products are generally recognized as relatively safe for human consumption, compared with synthetic drugs. Thus, the search for anti-AGE agents using herbal products has been increasing^52^. Many herbal products have potent anti-glycation activities, and these activities are similar or even stronger than aminoguanidine. For example, several polyphenols can inhibit the glycation process in vitro. Flavonoids are the major class of polyphenols. Anti-glycation properties of various flavonoids, such as kaempferol, genistein, quercitrin, and quercetin, have been reported^53-56^. Recently, we demonstrated a potent AGE breaking property of epicatechin in vitro and in vivo. This compound destroyed preformed glycated serum albumin in vitro and decreased AGE accumulation in retinal tissues of rats injected with exogenous AGE^42^. In the AGE structure, side chains attached to the pyrrole ring carbons are susceptible to nucleophilic attack^57^. Because C6 and C8 on the A-ring of epicatechin are nucleophilic^58^, epicatechin can attack and destroy the AGE cross-links.

## EFFECT of PHYSICAL EXERCISE on AGEs

Many previous reports have shown the ability of physical activity to improve glycemic control, with a consequent reduction of AGE accumulation in diabetic patients and during aging^36, 59^. In a rat model, 12 weeks of moderate physical exercise reduced the contents of CML and RAGE in aortic vessels^60^. Another study showed that rats subjected to treadmill exercise from late middle age to 35 months old had reduced AGE levels in cardiac tissues compared to age-matched control animals^61^. In human subjects, life-long trained athletes had 21% lower contents of AGE cross-links in the patellar tendon compared to age-matched untrained subjects^62^. Recently, we also showed the positive effect of regular exercise on the renal accumulation of AGEs. Specifically, regular exercise significantly prevented renal AGE deposition in D-galactose-induced aging rats. We also showed that treadmill exercise reduced CML accumulation and had retinoprotective effects in naturally-aged mice^63^.

Regular physical activity has beneficial contributions to physical capacity, hypertension, oxidative stress, and lipid metabolism^64, 65^. Especially, physical exercise effectively inhibits ROS generation and improves the activities of antioxidant enzymes^66^. The higher energy demands induced by physical exercise might reduce the pool of reactive intermediates available for glycation^21^. Because the protein glycation reaction is driven and accelerated by ROS, the inhibition of AGE formation by regular exercise may be the main mechanism of exercise-associated antioxidant activity. Additionally, AGE formation can be retarded or attenuated through efficient glycemic control^67^. Therefore, it can be assumed that regular physical exercise also can improve glycemic control, which attenuates the formation and accumulation of AGEs in tissues.

## CONCLUSION

In this review, we provide insights into the anti-glycation activities of herbal products and physical exercise. There is extensive scientific evidence documenting the accumulation of AGEs with aging and age-related diseases. Thus, we suggest that inhibiting the glycation process and removing existing glycation products may prolong the lifespan. In this sense, dietary herbal supplements or physiological exercise may be distinctly advantageous in reducing the burden of AGEs in our body.

## References

[1] Harman D (1981). The aging process. *Proc Natl Acad Sci U S A*.

[2] Gladyshev VN (2012). On the cause of aging and control of lifespan: heterogeneity leads to inevitable damage accumulation, causing aging; control of damage composition and rate of accumulation define lifespan. *Bioessays*.

[3] Gladyshev VN (2013). The origin of aging: imperfectness-driven non-random damage defines the aging process and control of lifespan. *Trends Genet*.

[4] Maillard L (1912). Action des acides amines sur les sucres; formation des melanoidines par voie methodique. *C R Acad Sci*.

[5] Sell DR, Monnier VM (2012). Molecular basis of arterial stiffening: role of glycation - a mini-review. *Gerontology*.

[6] Semba RD, Nicklett EJ, Ferrucci L (2010). Does accumulation of advanced glycation end products contribute to the aging phenotype?. *J Gerontol A Biol Sci Med Sci*.

[7] Nguyen HP, Katta R (2015). Sugar Sag: Glycation and the Role of Diet in Aging Skin. *Skin Therapy Lett*.

[8] Ahmed N, Thornalley PJ (2003). Quantitative screening of protein biomarkers of early glycation, advanced glycation, oxidation and nitrosation in cellular and extracellular proteins by tandem mass spectrometry multiple reaction monitoring. *Biochem Soc Trans*.

[9] Suji G, Sivakami S (2004). Glucose, glycation and aging. *Biogerontology*.

[10] Brownlee M (1995). Advanced protein glycosylation in diabetes and aging. *Annu Rev Med*.

[11] Baynes JW (1991). Role of oxidative stress in development of complications in diabetes. *Diabetes*.

[12] Yan SD, Yan SF, Chen X, Fu J, Chen M, Kuppusamy P (1995). Non-enzymatically glycated tau in Alzheimer's disease induces neuronal oxidant stress resulting in cytokine gene expression and release of amyloid beta-peptide. *Nat Med*.

[13] Giacco F, Brownlee M (2010). Oxidative stress and diabetic complications. *Circ Res*.

[14] Peng X, Ma J, Chen F, Wang M (2011). Naturally occurring inhibitors against the formation of advanced glycation end-products. *Food Funct*.

[15] Sadowska-Bartosz I, Bartosz G (2016). Effect of glycation inhibitors on aging and age-related diseases. *Mech Ageing Dev*.

[16] Kim JW, No JK, Ikeno Y, Yu BP, Choi JS, Yokozawa T (2002). Age-related changes in redox status of rat serum. *Arch Gerontol Geriatr*.

[17] Radak Z, Chung HY, Naito H, Takahashi R, Jung KJ, Kim HJ (2004). Age-associated increase in oxidative stress and nuclear factor kappaB activation are attenuated in rat liver by regular exercise. *FASEB J*.

[18] Asghar M, George L, Lokhandwala MF (2007). Exercise decreases oxidative stress and inflammation and restores renal dopamine D1 receptor function in old rats. *Am J Physiol Renal Physiol*.

[19] Navarro A, Gomez C, Lopez-Cepero JM, Boveris A (2004). Beneficial effects of moderate exercise on mice aging: survival, behavior, oxidative stress, and mitochondrial electron transfer. *Am J Physiol Regul Integr Comp Physiol*.

[20] Gomez-Cabrera MC, Domenech E, Vina J (2008). Moderate exercise is an antioxidant: upregulation of antioxidant genes by training. *Free Radic Biol Med*.

[21] Boor P, Celec P, Behuliak M, Grancic P, Kebis A, Kukan M (2009). Regular moderate exercise reduces advanced glycation and ameliorates early diabetic nephropathy in obese Zucker rats. *Metabolism*.

[22] Boule NG, Haddad E, Kenny GP, Wells GA, Sigal RJ (2001). Effects of exercise on glycemic control and body mass in type 2 diabetes mellitus: a meta-analysis of controlled clinical trials. *JAMA*.

[23] Oudes AJ, Herr CM, Olsen Y, Fleming JE (1998). Age-dependent accumulation of advanced glycation end-products in adult Drosophila melanogaster. *Mech Ageing Dev*.

[24] Schlotterer A, Kukudov G, Bozorgmehr F, Hutter H, Du X, Oikonomou D (2009). C. elegans as model for the study of high glucose-mediated life span reduction. *Diabetes*.

[25] Ott C, Jacobs K, Haucke E, Navarrete Santos A, Grune T, Simm A (2014). Role of advanced glycation end products in cellular signaling. *Redox Biol*.

[26] Schalkwijk CG, Miyata T (2012). Early- and advanced non-enzymatic glycation in diabetic vascular complications: the search for therapeutics. *Amino Acids*.

[27] Dalal M, Sun K, Cappola AR, Ferrucci L, Crasto C, Fried LP (2011). Relationship of serum fibroblast growth factor 23 with cardiovascular disease in older community-dwelling women. *Eur J Endocrinol*.

[28] Pageon H, Zucchi H, Rousset F, Monnier VM, Asselineau D (2014). Skin aging by glycation: lessons from the reconstructed skin model. *Clin Chem Lab Med*.

[29] Ulrich P, Cerami A (2001). Protein glycation, diabetes, and aging. *Recent Prog Horm Res*.

[30] Yao D, Taguchi T, Matsumura T, Pestell R, Edelstein D, Giardino I (2007). High glucose increases angiopoietin-2 transcription in microvascular endothelial cells through methylglyoxal modification of mSin3A. *J Biol Chem*.

[31] Wu L, Juurlink BH (2002). Increased methylglyoxal and oxidative stress in hypertensive rat vascular smooth muscle cells. *Hypertension*.

[32] Bakala H, Ladouce R, Baraibar MA, Friguet B (2013). Differential expression and glycative damage affect specific mitochondrial proteins with aging in rat liver. *Biochim Biophys Acta*.

[33] Reeg S, Grune T (2015). Protein Oxidation in Aging: Does It Play a Role in Aging Progression?. *Antioxid Redox Signal*.

[34] Boulanger E, Wautier MP, Wautier JL, Boval B, Panis Y, Wernert N (2002). AGEs bind to mesothelial cells via RAGE and stimulate VCAM-1 expression. *Kidney Int*.

[35] Roca F, Grossin N, Chassagne P, Puisieux F, Boulanger E (2014). Glycation: the angiogenic paradox in aging and age-related disorders and diseases. *Ageing Res Rev*.

[36] Omsland TK, Bangstad HJ, Berg TJ, Kolset SO (2006). Advanced glycation end products and hyperglycaemia. *Tidsskr Nor Laegeforen*.

[37] Grossin N, Auger F, Niquet-Leridon C, Durieux N, Montaigne D, Schmidt AM (2015). Dietary CML-enriched protein induces functional arterial aging in a RAGE-dependent manner in mice. *Mol Nutr Food Res*.

[38] Stopper H, Schinzel R, Sebekova K, Heidland A (2003). Genotoxicity of advanced glycation end products in mammalian cells. *Cancer Lett*.

[39] Peppa M, Brem H, Ehrlich P, Zhang JG, Cai W, Li Z (2003). Adverse effects of dietary glycotoxins on wound healing in genetically diabetic mice. *Diabetes*.

[40] Uribarri J, Peppa M, Cai W, Goldberg T, Lu M, He C (2003). Restriction of dietary glycotoxins reduces excessive advanced glycation end products in renal failure patients. *J Am Soc Nephrol*.

[41] Huebschmann AG, Regensteiner JG, Vlassara H, Reusch JE (2006). Diabetes and advanced glycoxidation end products. *Diabetes Care*.

[42] Kim J, Kim CS, Moon MK, Kim JS (2015). Epicatechin breaks preformed glycated serum albumin and reverses the retinal accumulation of advanced glycation end products. *Eur J Pharmacol*.

[43] Brownlee M, Vlassara H, Kooney A, Ulrich P, Cerami A (1986). Aminoguanidine prevents diabetes-induced arterial wall protein cross-linking. *Science*.

[44] Lo TW, Selwood T, Thornalley PJ (1994). The reaction of methylglyoxal with aminoguanidine under physiological conditions and prevention of methylglyoxal binding to plasma proteins. *Biochem Pharmacol*.

[45] Thornalley PJ, Yurek-George A, Argirov OK (2000). Kinetics and mechanism of the reaction of aminoguanidine with the alpha-oxoaldehydes glyoxal, methylglyoxal, and 3-deoxyglucosone under physiological conditions. *Biochem Pharmacol*.

[46] Thornalley PJ (2003). Use of aminoguanidine (Pimagedine) to prevent the formation of advanced glycation endproducts. *Arch Biochem Biophys*.

[47] Suji G, Sivakami S (2006). DNA damage by free radical production by aminoguanidine. *Ann N Y Acad Sci*.

[48] Tilton RG, Chang K, Hasan KS, Smith SR, Petrash JM, Misko TP (1993). Prevention of diabetic vascular dysfunction by guanidines. Inhibition of nitric oxide synthase versus advanced glycation end-product formation. *Diabetes*.

[49] Turgut F, Bolton WK (2010). Potential new therapeutic agents for diabetic kidney disease. *Am J Kidney Dis*.

[50] Wolffenbuttel BH, Boulanger CM, Crijns FR, Huijberts MS, Poitevin P, Swennen GN (1998). Breakers of advanced glycation end products restore large artery properties in experimental diabetes. *Proc Natl Acad Sci U S A*.

[51] Sugiyama S, Miyata T, Ueda Y, Tanaka H, Maeda K, Kawashima S (1998). Plasma levels of pentosidine in diabetic patients: an advanced glycation end product. *J Am Soc Nephrol*.

[52] Lee HS, Jung SH, Yun BS, Lee KW (2007). Isolation of chebulic acid from Terminalia chebula Retz. and its antioxidant effect in isolated rat hepatocytes. *Arch Toxicol*.

[53] Lv L, Shao X, Chen H, Ho CT, Sang S (2011). Genistein inhibits advanced glycation end product formation by trapping methylglyoxal. *Chem Res Toxicol*.

[54] Bournival J, Francoeur MA, Renaud J, Martinoli MG (2012). Quercetin and sesamin protect neuronal PC12 cells from high-glucose-induced oxidation, nitrosative stress, and apoptosis. *Rejuvenation Res*.

[55] Sadowska-Bartosz I, Galiniak S, Bartosz G (2014). Kinetics of glycoxidation of bovine serum albumin by methylglyoxal and glyoxal and its prevention by various compounds. *Molecules*.

[56] Xie Y, Chen X (2013). Structures required of polyphenols for inhibiting advanced glycation end products formation. *Curr Drug Metab*.

[57] Baba S, Osakabe N, Natsume M, Muto Y, Takizawa T, Terao J (2001). In vivo comparison of the bioavailability of (+)-catechin, (-)-epicatechin and their mixture in orally administered rats. *J Nutr*.

[58] Wang L, Tian W, Uwais Z, Li G, Li H, Guan R (2014). AGE-breaker ALT-711 plus insulin could restore erectile function in streptozocin-induced type 1 diabetic rats. *J Sex Med*.

[59] Panteleeva IG, Rogozkin VA (2001). Effect of physical load on serum protein glycation in rats with induced diabetes. *Ross Fiziol Zh Im I M Sechenova*.

[60] Gu Q, Wang B, Zhang XF, Ma YP, Liu JD, Wang XZ (2014). Contribution of receptor for advanced glycation end products to vasculature-protecting effects of exercise training in aged rats. *Eur J Pharmacol*.

[61] Wright KJ, Thomas MM, Betik AC, Belke D, Hepple RT (2014). Exercise training initiated in late middle age attenuates cardiac fibrosis and advanced glycation end-product accumulation in senescent rats. *Exp Gerontol*.

[62] Couppe C, Svensson RB, Grosset JF, Kovanen V, Nielsen RH, Olsen MR (2014). Life-long endurance running is associated with reduced glycation and mechanical stress in connective tissue. *Age (Dordr)*.

[63] Kim CS, Park S, Chun Y, Song W, Kim HJ, Kim J (2015). Treadmill Exercise Attenuates Retinal Oxidative Stress in Naturally-Aged Mice: An Immunohistochemical Study. *Int J Mol Sci*.

[64] Pechter U, Maaroos J, Mesikepp S, Veraksits A, Ots M (2003). Regular low-intensity aquatic exercise improves cardio-respiratory functional capacity and reduces proteinuria in chronic renal failure patients. *Nephrol Dial Transplant*.

[65] Moinuddin I, Leehey DJ (2008). A comparison of aerobic exercise and resistance training in patients with and without chronic kidney disease. *Adv Chronic Kidney Dis*.

[66] Coelho BL, Rocha LG, Scarabelot KS, Scheffer DL, Ronsani MM, Silveira PC (2010). Physical exercise prevents the exacerbation of oxidative stress parameters in chronic kidney disease. *J Ren Nutr*.

[67] Turk Z, Misur I, Turk N, Benko B (1999). Rat tissue collagen modified by advanced glycation: correlation with duration of diabetes and glycemic control. *Clin Chem Lab Med*.

[68] Hu S, He W, Liu Z, Xu H, Ma G (2013). The accumulation of the glycoxidation product N(epsilon)-carboxymethyllysine in cardiac tissues with age, diabetes mellitus and coronary heart disease. *Tohoku J Exp Med*.

[69] Albon J, Karwatowski WS, Avery N, Easty DL, Duance VC (1995). Changes in the collagenous matrix of the aging human lamina cribrosa. *Br J Ophthalmol*.

[70] Bellmunt MJ, Portero M, Pamplona R, Cosso L, Odetti P, Prat J (1995). Evidence for the Maillard reaction in rat lung collagen and its relationship with solubility and age. *Biochim Biophys Acta*.

[71] Nowotny K, Grune T (2014). Degradation of oxidized and glycoxidized collagen: role of collagen cross-linking. *Arch Biochem Biophys*.

[72] van Deemter M, Ponsioen TL, Bank RA, Snabel JM, van der Worp RJ, Hooymans JM (2009). Pentosidine accumulates in the aging vitreous body: a gender effect. *Exp Eye Res*.

[73] Matsumine M, Shibata N, Ishitani K, Kobayashi M, Ohta H (2008). Pentosidine accumulation in human oocytes and their correlation to age-related apoptosis. *Acta Histochem Cytochem*.

[74] Sivan SS, Tsitron E, Wachtel E, Roughley P, Sakkee N, van der Ham F (2006). Age-related accumulation of pentosidine in aggrecan and collagen from normal and degenerate human intervertebral discs. *Biochem J*.

[75] Verzijl N, DeGroot J, Oldehinkel E, Bank RA, Thorpe SR, Baynes JW (2000). Age-related accumulation of Maillard reaction products in human articular cartilage collagen. *Biochem J*.

